# Supplementary material for: Developing a Predictive Grading Model for Children with Gliomas Based on Diffusion Kurtosis Imaging Metrics: Accuracy and Clinical Correlations with Patient Survival
Source: Cancers (Basel). 2022 Sep 29;14(19):4778. doi: 10.3390/cancers14194778 (PMC9563289; doi:10.3390/cancers14194778)
Supplement: Supplementary file 1 [file cancers-14-04778-s001.zip › cancers-1857001 File S1.pdf]

#DKI CODE PREDICTION MODEL R

#This R code was used to build and test the grading accuracy of the prediction models based on the DKI sequence. Three predictive models were built and their accuracy was tested with ROC analysis.
We provide this code for free use under the terms and conditions of the Creative Commons Attribution (CC BY) license (https://creativecommons.org/licenses/by/4.0/).
If you use this code for your scientific research, please cite this work:

Voicu, I.P.; Napolitano, A.; Caulo, M.; Dotta, F.; Piccirilli, E.; Vinci, M.; Diomedi-Camassei, F.; Lattavo, L.; Carboni, A.; Miele, E.; et al. Developing a Predictive Grading Model for Children with Gliomas Based on Diffusion Kurtosis Imaging Metrics: Accuracy and Clinical Correlations with Patient Survival. Cancers 2022, 14, 4778. https://doi.org/10.3390/cancers14194778

#After opening R, we load the packages that will be needed for the analyses.

```
library("pROC")
library("glmnet")
library("sjmisc")
library("magrittr")
library("plyr")
library("readxl")
library("mlbench")
library("caret")
library("psych")
library("sjlabelled")
library("haven")
```

#then, we import the data provided on Table S1

```
dki_simple <- read_excel("DKI.xls")
```

#after importing the data, we test the grading accuracy of the single kurtosis-derived metrics with ROC analysis based on the package pROC

```
ak <- roc(dki_simple$Tumor_grade_0_1,dki_simple$`Axial Kurtosis`)
auc(ak)
mk <- roc(dki_simple$Tumor_grade_0_1,dki_simple$`Mean Kurtosis`)
rk <- roc(dki_simple$Tumor_grade_0_1,dki_simple$`Radial Kurtosis`)
fa <- roc(dki_simple$Tumor_grade_0_1,dki_simple$`Fractional Anisotropy`)
adc <- roc(dki_simple$Tumor_grade_0_1,dki_simple$`Mean diffusivity-ADC`)
```

#we calculate the AUC of the single metrics

```
auc(mk)
auc(ak)
auc(rk)
```

```
auc(fa)
auc(adc)
```

#then, we plot the ROC curves for each metric. In our work we chose to plot the curves together by selecting add=T

```
plot.roc(ak,col="red")
plot.roc(mk,col = "green",add=T)
plot.roc(rk,col="yellow",add=T)
plot.roc(fa,col="light blue")
plot.roc(ak,col="red",add=T)
plot.roc(mk,col = "green",add=T)
plot.roc(rk,col="yellow",add=T)
plot.roc(adc,col="blue",add=T)
```

#after calculating the AUC for each kurtosis metric, we build and test the predictive accuracy of a penalized Elasticnet regression model based on data from all kurtosis metrics. (Elasticnet is a combination of LASSO and RIDGE penalties for regression analyses).

```
x<-as.matrix(dki_simple [,13:17])
y<-as.matrix(dki_simple[,6])
fit_elastic_dki<-glmnet(x,y,family="binomial",alpha=0.5, lambda=0.001)
predict.glmnet(fit_elastic_dki,type="coefficients")
predictions<-predict(fit_elastic_dki,x,type="response")
model_accuracy<-roc(dki_simple$Tumor_grade_0_1,predictions)
auc(model_accuracy)
plot.roc(model_accuracy,col="green")
coords(model_accuracy,x="best",best.method = "closest.topleft",transpose=TRUE)
coords(model_accuracy,x="best",best.method = "youden",transpose=TRUE)
sensitivities<-ci.se(model_accuracy)
plot(sensitivities,type = "s",no.roc = FALSE,col = "orange")
plot.roc(model_accuracy,col="green",add = TRUE)
plot(sensitivities,type = "s",no.roc = FALSE,col = "red",auc.polygon.border=NULL)
plot.roc(model_accuracy,col="blue",add = TRUE)
predict.glmnet(fit_elastic_dki,type="coefficients")
show(predictions)
```

#From the original Dki diffusion sequence, both DTI- specific metrics such as FA and ADC-MD and DKI-specific metrics such as MK, RK, AK can be obtained. After we previously built a combined predictive model based on all metrics, we now build and test a model based on DTI metrics only.

```
x_dti<-as.matrix(dki_simple [,16:17])
y_dti<-as.matrix(dki_simple [,6])
```

```
fit_elastic_dti<-glmnet(x_dti,y_dti,family="binomial",alpha=0.5, lambda=0.001)
predict.glmnet(fit_elastic_dti,type="coefficients")
predictions_dti<-predict(fit_elastic_dti,x_dti,type="response")
dti_model_acc<-roc(y_dti,predictions_dti)
auc(dti_model_acc)
roc.test(dti_model_acc,model_accuracy)

#Then, we build and test a model baed on DKI metrics only.

x_dki<-as.matrix(dki_simple [,13:15])
y_dki<-as.matrix(dki_simple [,6])
fit_elastic_dki<-glmnet(x_dki,y_dki,family="binomial",alpha=0.5, lambda=0.001)
predict.glmnet(fit_elastic_dki,type="coefficients")
predictions_dki<-predict(fit_elastic_dki,x_dki,type="response")
dki_model_acc<-roc(y_dki,predictions_dki)
auc(dki_model_acc)
prediction_coefficient <- data.frame(predictions)

#Finally, we plot the AUC of the 3 predictive models.

dki_new <- cbind(dki_simple,prediction_coefficient)
dki_new$s0 <- as.numeric(dki_new$s0)
f<-as_factor(dki_new$Tumor_grade_0_1)
model_accuracy<-roc(f,dki_new$s0)
plot.roc(dki_model_acc,col=y)
plot.roc(model_accuracy,add=T)
plot.roc(dti_model_acc,add = T)
plot.roc(dki_model_acc,col="green")
plot.roc(model_accuracy,add=T,col="blue")
plot.roc(dti_model_acc,add = T,col="red")
ci(dti_model_acc)
ci(dki_model_acc)
```
