# Supplementary material for: Developing a Predictive Grading Model for Children with Gliomas Based on Diffusion Kurtosis Imaging Metrics: Accuracy and Clinical Correlations with Patient Survival
Source: Cancers (Basel). 2022 Sep 29;14(19):4778. doi: 10.3390/cancers14194778 (PMC9563289; doi:10.3390/cancers14194778)
Supplement: Supplementary file 1 [file cancers-14-04778-s001.zip › cancers-1857001-Figures.pdf]

Supplementary Materials

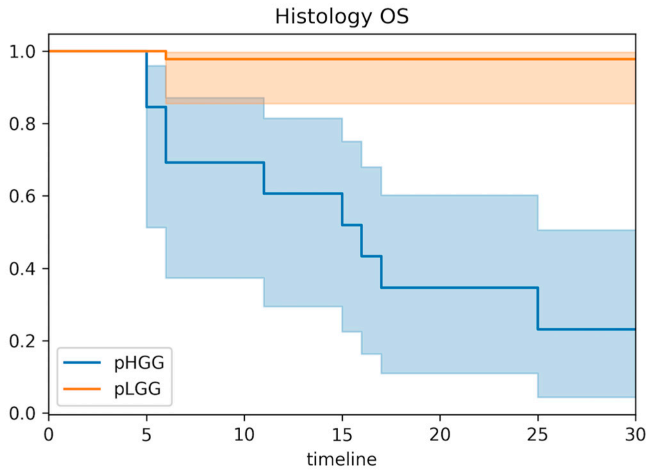

Figure S1. Correlations between high or low grade glioma histology and patient OS.

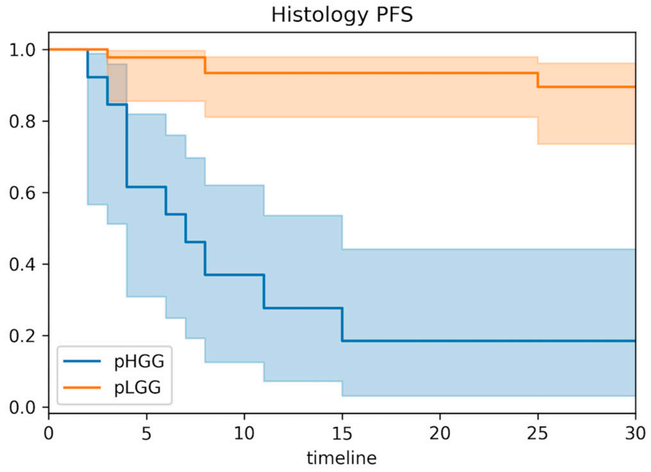

Figure S2. Correlations between high or low grade glioma histology and patient PFS.
